# Supplementary material for: Unveiling the Bioactive Potential of the Invasive Jellyfish Phyllorhiza punctata Through Integrative Transcriptomic and Proteomic Analyses
Source: Biomolecules. 2025 Aug 4;15(8):1121. doi: 10.3390/biom15081121 (PMC12383608; doi:10.3390/biom15081121)
Supplement: Supplementary file 1 [file biomolecules-15-01121-s001.zip › Suplementary Material/FileS3/PEPFOLD4/XP_065054978_16065/clusters.html]

 PEP-FOLD clusters


Download archive of all models  here   

|  |  |  |
| --- | --- | --- |
| # | file | sOPEP |
| # cluster 1 - Size: 18 |  |  |
|  | XP\_065054978\_16065-model1 | -8.23006 |
|  | XP\_065054978\_16065-model1.1 | -7.72727 |
|  | XP\_065054978\_16065-model1.2 | -7.71488 |
|  | XP\_065054978\_16065-model1.3 | -7.66117 |
|  | XP\_065054978\_16065-model1.4 | -7.58137 |
|  | XP\_065054978\_16065-model1.5 | -7.52473 |
|  | XP\_065054978\_16065-model1.6 | -7.45075 |
|  | XP\_065054978\_16065-model1.7 | -7.40138 |
|  | XP\_065054978\_16065-model1.8 | -7.21444 |
|  | XP\_065054978\_16065-model1.9 | -7.20868 |
|  | XP\_065054978\_16065-model1.10 | -7.1757 |
|  | XP\_065054978\_16065-model1.11 | -7.16169 |
|  | XP\_065054978\_16065-model1.12 | -7.11348 |
|  | XP\_065054978\_16065-model1.13 | -7.06735 |
|  | XP\_065054978\_16065-model1.14 | -6.98076 |
|  | XP\_065054978\_16065-model1.15 | -6.95061 |
|  | XP\_065054978\_16065-model1.16 | -6.89274 |
|  | XP\_065054978\_16065-model1.17 | -6.50749 |
| # cluster 2 - Size: 15 |  |  |
|  | XP\_065054978\_16065-model2 | -7.68882 |
|  | XP\_065054978\_16065-model2.1 | -7.6202 |
|  | XP\_065054978\_16065-model2.2 | -7.5236 |
|  | XP\_065054978\_16065-model2.3 | -7.4035 |
|  | XP\_065054978\_16065-model2.4 | -7.31178 |
|  | XP\_065054978\_16065-model2.5 | -7.2853 |
|  | XP\_065054978\_16065-model2.6 | -7.27448 |
|  | XP\_065054978\_16065-model2.7 | -7.27139 |
|  | XP\_065054978\_16065-model2.8 | -7.21905 |
|  | XP\_065054978\_16065-model2.9 | -7.13847 |
|  | XP\_065054978\_16065-model2.10 | -7.12704 |
|  | XP\_065054978\_16065-model2.11 | -7.06452 |
|  | XP\_065054978\_16065-model2.12 | -7.06413 |
|  | XP\_065054978\_16065-model2.13 | -6.9387 |
|  | XP\_065054978\_16065-model2.14 | -6.92932 |
| # cluster 3 - Size: 21 |  |  |
|  | XP\_065054978\_16065-model3 | -7.65622 |
|  | XP\_065054978\_16065-model3.1 | -7.45493 |
|  | XP\_065054978\_16065-model3.2 | -7.44989 |
|  | XP\_065054978\_16065-model3.3 | -7.36891 |
|  | XP\_065054978\_16065-model3.4 | -7.30896 |
|  | XP\_065054978\_16065-model3.5 | -7.29294 |
|  | XP\_065054978\_16065-model3.6 | -7.16592 |
|  | XP\_065054978\_16065-model3.7 | -7.16109 |
|  | XP\_065054978\_16065-model3.8 | -7.16072 |
|  | XP\_065054978\_16065-model3.9 | -7.14773 |
|  | XP\_065054978\_16065-model3.10 | -7.10887 |
|  | XP\_065054978\_16065-model3.11 | -7.10327 |
|  | XP\_065054978\_16065-model3.12 | -7.09329 |
|  | XP\_065054978\_16065-model3.13 | -7.08875 |
|  | XP\_065054978\_16065-model3.14 | -7.05183 |
|  | XP\_065054978\_16065-model3.15 | -7.02352 |
|  | XP\_065054978\_16065-model3.16 | -6.98684 |
|  | XP\_065054978\_16065-model3.17 | -6.95265 |
|  | XP\_065054978\_16065-model3.18 | -6.80236 |
|  | XP\_065054978\_16065-model3.19 | -6.66708 |
|  | XP\_065054978\_16065-model3.20 | -6.26792 |
| # cluster 4 - Size: 7 |  |  |
|  | XP\_065054978\_16065-model4 | -7.56936 |
|  | XP\_065054978\_16065-model4.1 | -7.56335 |
|  | XP\_065054978\_16065-model4.2 | -7.38497 |
|  | XP\_065054978\_16065-model4.3 | -7.36506 |
|  | XP\_065054978\_16065-model4.4 | -7.35792 |
|  | XP\_065054978\_16065-model4.5 | -6.93238 |
|  | XP\_065054978\_16065-model4.6 | -6.83448 |
| # cluster 5 - Size: 8 |  |  |
|  | XP\_065054978\_16065-model5 | -7.46841 |
|  | XP\_065054978\_16065-model5.1 | -7.34473 |
|  | XP\_065054978\_16065-model5.2 | -7.29479 |
|  | XP\_065054978\_16065-model5.3 | -7.28404 |
|  | XP\_065054978\_16065-model5.4 | -7.15365 |
|  | XP\_065054978\_16065-model5.5 | -7.01959 |
|  | XP\_065054978\_16065-model5.6 | -6.64734 |
|  | XP\_065054978\_16065-model5.7 | -6.16204 |
| # cluster 6 - Size: 1 |  |  |
|  | XP\_065054978\_16065-model6 | -7.4375 |
| # cluster 7 - Size: 9 |  |  |
|  | XP\_065054978\_16065-model7 | -7.249 |
|  | XP\_065054978\_16065-model7.1 | -7.21913 |
|  | XP\_065054978\_16065-model7.2 | -7.14746 |
|  | XP\_065054978\_16065-model7.3 | -6.95706 |
|  | XP\_065054978\_16065-model7.4 | -6.93118 |
|  | XP\_065054978\_16065-model7.5 | -6.91134 |
|  | XP\_065054978\_16065-model7.6 | -6.81437 |
|  | XP\_065054978\_16065-model7.7 | -6.80064 |
|  | XP\_065054978\_16065-model7.8 | -6.79948 |
| # cluster 8 - Size: 1 |  |  |
|  | XP\_065054978\_16065-model8 | -7.20902 |
| # cluster 9 - Size: 5 |  |  |
|  | XP\_065054978\_16065-model9 | -7.20007 |
|  | XP\_065054978\_16065-model9.1 | -7.08922 |
|  | XP\_065054978\_16065-model9.2 | -7.01799 |
|  | XP\_065054978\_16065-model9.3 | -6.82083 |
|  | XP\_065054978\_16065-model9.4 | -6.52103 |
| # cluster 10 - Size: 9 |  |  |
|  | XP\_065054978\_16065-model10 | -7.16358 |
|  | XP\_065054978\_16065-model10.1 | -7.1627 |
|  | XP\_065054978\_16065-model10.2 | -7.07113 |
|  | XP\_065054978\_16065-model10.3 | -6.98482 |
|  | XP\_065054978\_16065-model10.4 | -6.79604 |
|  | XP\_065054978\_16065-model10.5 | -6.79174 |
|  | XP\_065054978\_16065-model10.6 | -6.75897 |
|  | XP\_065054978\_16065-model10.7 | -6.58474 |
|  | XP\_065054978\_16065-model10.8 | -6.33101 |
| # cluster 11 - Size: 2 |  |  |
|  | XP\_065054978\_16065-model11 | -6.90187 |
|  | XP\_065054978\_16065-model11.1 | -6.4106 |
| # cluster 12 - Size: 1 |  |  |
|  | XP\_065054978\_16065-model12 | -6.43341 |
| # cluster 13 - Size: 1 |  |  |
|  | XP\_065054978\_16065-model13 | -6.33276 |
| # cluster 14 - Size: 2 |  |  |
|  | XP\_065054978\_16065-model14 | -6.19435 |
|  | XP\_065054978\_16065-model14.1 | -5.99563 |
|  |  |  |
